# Screening for subclinical rheumatic heart disease: addressing borderline disease in a real-world setting

**DOI:** 10.1093/ehjopen/oeab041

**Published:** 2021-12-27

**Authors:** Luke D Hunter, Alfonso J K Pecoraro, Anton F Doubell, Mark J Monaghan, Guy W Lloyd, Carl J Lombard, Philip G Herbst

**Affiliations:** 1 Division of Cardiology, Department of Medicine, Faculty of Medicine and Health Sciences, University of Stellenbosch and Tygerberg Academic Hospital, Francie van Zijl Drive, Tygerberg, 7505, Cape Town, South Africa;; 2 Department of Cardiology, King's College Hospital, Denmark Hill, London, SE5 9RS, United Kingdom;; 3 Echocardiography Laboratory, Barts Heart Centre, St Bartholomew’s Hospital,West Smithfield, London EC1A 7BE, United Kingdom;; 4 Institute of Cardiovascular Sciences, University College London, 62 Huntley St,WC1E 6DD, London, United Kingdom;; 5 William Harvey Research Institute Barts & The London School of Medicine & Dentistry, Queen Mary University of London, Charterhouse Square, London, EC1M 6BQ, United Kingdom;; 6 Division of Epidemiology and Biostatistics, Department of Global Health Faculty of Medicine and Health Sciences, Stellenbosch University, Francie van Zijl Drive, Tygerberg, Cape Town,7505, South Africa; and; 7 Biostatistics Unit, South African Medical Research Council, Cape Town, South Africa

**Keywords:** Rheumatic heart disease, Screening echocardiography

## Abstract

**Aims:**

The World Heart Federation (WHF) criteria identify a large borderline rheumatic heart disease (RHD) category that has hampered the implementation of population-based screening. Inter-scallop separations (ISS) of the posterior mitral valve leaflet, a recently described normal variant of the mitral valve, appears to be an important cause of mild mitral regurgitation (MR) leading to misclassification of cases as WHF ‘borderline RHD’. This study aims to report the findings of the Echo in Africa project, a large-scale RHD screening project in South Africa and determine what proportion of borderline cases would be re-classified as normal if there were a systematic identification of ISS-related MR.

**Methods and results:**

A prospective cross-sectional study of underserved secondary schools in the Western Cape was conducted. Participants underwent a screening study with a handheld (HH) ultrasound device. Children with an abnormal HH study were re-evaluated with a portable laptop echocardiography machine. A mechanistic evaluation was applied in cases with isolated WHF ‘pathological’ MR (WHF ‘borderline RHD’). A total of 5255 participants (mean age 15± years) were screened. A total of 3439 (65.8%) were female. Forty-nine cases of WHF ‘definite RHD’ [9.1 cases/1000 (95% confidence interval, CI, 6.8–12.1 cases/1000)] and 104 cases of WHF ‘borderline RHD’ [19.5 cases/1000 (95% CI, 16.0–23.7 cases/1000)] were identified. Inter-scallop separations-related MR was the underlying mechanism of MR in 48/68 cases classified as WHF ‘borderline RHD’ with isolated WHF ‘pathological’ MR (70.5%).

**Conclusion:**

In a real-world, large-scale screening project, the adoption of a mechanistic evaluation based on the systematic identification of ISS-related MR markedly reduced the number of WHF ‘screen-positive’ cases misclassified as WHF ‘borderline RHD’. Implementing strategies that reduce this misclassification could reduce the cost- and labour burden on large-scale RHD screening programmes.

## Introduction

Screening echocardiography is recognized as the diagnostic investigation of choice for the identification of rheumatic heart disease (RHD) amongst asymptomatic children.^1^ The World Heart Federation (WHF) criteria were developed to standardize the reporting and diagnostic approach to ‘subclinical’ RHD.^1^ The WHF criteria have reinvigorated RHD research in Sub-Saharan Africa^2–9^ and have galvanized amendments to official RHD health policy.^10^ Unfortunately, the criteria create a large borderline group; a diagnostic category reserved for screened cases demonstrating some, but not all of the required criteria for a definite diagnosis.^1^

The majority of cases in the ‘borderline’ RHD category are identified with isolated, so-called ‘pathological’ mitral regurgitation (MR).^11^ Longitudinal study of this heterogeneous cohort has yet to provide conclusive evidence that its identification and treatment confers any prognostic benefit and raises the question of whether this even represents a predominantly RHD group.^8^^,^^11–15^ Through the Echo in Africa (EIA) project, we have recently identified and described inter-scallop separations (ISS) of the posterior mitral valve leaflet (PMVL), a common, normal finding of the mitral valve (MV) and an important cause of isolated WHF ‘pathological’ MR.^11^ The adoption of an MR assessment that determines the underlying mechanism of MR through its increased specificity for causative aetiologies, has the potential to reduce the number of ‘screen-positive’ WHF cases misclassified as rheumatic with the promise of reducing cost- and labour burden on large-scale RHD screening programmes. In the present study, we report the 5-year findings of the EIA project (2014–18) and assess the impact that a systematic identification of ISS-related ‘pathological’ MR would have on the number of patients allocated to the WHF ‘borderline’ RHD category.

## Methods

### Study design and participants

The EIA project is a collaborative initiative between SUNHEART [a non-profit organisation established by the Division of Cardiology at Tygerberg Academic Hospital (TBH)] and the British Society of Echocardiography (BSE). The EIA team conducted a prospective cross-sectional study in school children attending secondary state (public) schools in the Western Cape, South Africa. Ethical approval was obtained through the University of Stellenbosch and the Department of Education in the Western Cape, respectively (N14/04/038). The project’s footprint spans three adjacent district municipalities, namely the City of Cape Town- (six schools), Drakenstein- (two schools), and Stellenbosch municipalities (two schools). Schools in low-income areas within each district municipality were selected based on a national quintile score—a standardized poverty indicator that reflects the income, unemployment, and level of education within each community.^16^ Informed parental/guardian consent was required before study enrolment. Annual EIA screening camps, typically lasting 4 weeks were scheduled. Each week, roughly 10 BSE-accredited sonographers provided echocardiographic support for the project. All sonographers were required to complete a distance learning module on rheumatic valve disease morphology and evaluation. After an initial hands-on training period, study investigators provided ongoing on-site tuition and support to all screeners for the duration of the screening programme.

### Echocardiographic evaluation

In 2014 and 2015, all enrolled study participants were screened in a purpose-renovated facility at TBH. All transthoracic echocardiogram (TTE) studies were performed by a cardiologist experienced in the assessment of valve disease or a BSE-accredited sonographer under the guidance of such a cardiologist. Participants were screened with a portable handheld device [HH; General Electric (GE™) V-scan] using a pre-defined study protocol that has been previously described.^17^ This was followed by a comprehensive validation TTE study using a GE™ Vivid I portable laptop machine with a 2- to 3.6-MHz transducer probe (GE™ 3S). The validation study was performed according to the current BSE guideline for a standard adult TTE.^18^ It was supplemented by an MV evaluation aimed at extracting the more specific information required by the WHF and a ‘Carpentier-style’ mechanistic evaluation that has been previously described.^11^

After the first 2 years of screening, the project shifted focus to become a community-based programme providing HH echocardiographic screening to children at their respective schools. Only children with an abnormal screening HH study [defined as an MR jet length ≥1.5 cm, aortic regurgitation (AR) jet length ≥0.5 cm, or any WHF morphological features of RHD, congenital, or acquired heart disease] underwent a comprehensive study at TBH as previously described. Each screened case was reviewed and reported on-site by an expert, experienced in the echocardiographic evaluation of RHD (L.D.H., A.J.K.P., A.F.D., M.J.M., G.W.L., and P.G.H.).

### Data management and analysis strategy

All study participant data were deidentified and entered into a Google™ Cloud Platform service (Google™ Sheets). The V-scan images from each study were downloaded to a study personal computer and accessed using GE™ Gateway software. The comprehensive echocardiographic studies were loaded onto an image viewing network (GE™ ECHOPAC). Cases of congenital heart disease were excluded from further analysis for RHD. After consensus review, comprehensive scans were categorised according to the WHF criteria as having WHF-‘normal’, WHF-‘borderline’, or WHF-‘definite RHD’. A ‘Carpentier-style’ evaluation was used to create five pre-defined mechanistic groups of MR including (i) MV prolapse or prolapse spectrum, (ii) rheumatic based on the presence of pseudo-prolapse, (iii) congenital anterior mitral valve leaflet (AMVL) cleft and fenestration, (iv) ISS-related MR, and (v) an indeterminate category.^11^ In cases where unanimity regarding diagnosis could not be obtained, an independent reviewer was appointed to adjudicate with the view to reaching a final diagnosis.

### Statistical analysis

Data were entered into an Excel 2019 database (Microsoft), and statistical analysis was conducted in Stata 15 (StataCorp 2017). Descriptive statistical analysis was undertaken, and the prevalence of WHF ‘screen-detected’ RHD estimated with 95% confidence intervals (CIs). A *post hoc* weighted analysis was performed to more accurately assess the prevalence of RHD in the studied population. Overall survey weights were calculated to reflect the population of potential underserved children in the three district municipalities. The overall weights were based on the fraction of school children sampled from the underserved population at a school level in a district municipality and secondly the fraction of children screened in the schools that were sampled. This weighting was done at a district municipality level, and each child was allocated the same weight-based on the area. The reference data and *post hoc* weights are included in the Supplementary material online, *Addendum A*. Basic descriptive tables were done for describing the characteristics of the WHF- and mechanistic MR assessment and 95% CIs were calculated for the prevalence of the RHD categories.

## Results

The descriptive data and estimated prevalence of echocardiographic RHD are presented in *Table 1*. A total of 5225 secondary school children (aged 13–19) were enrolled in the study. The mean age of screened schoolchildren was 15 years (standard deviation, 2 years) of which 3439 (65.8%) were female. A total of 49 WHF ‘definite RHD’ and 104 ‘borderline RHD’ cases were detected by echocardiography. The overall estimated prevalence of WHF-‘definite RHD’, -‘borderline’, and -total were respectively 9.1 cases/1000 population (95% CI, 6.8–12.1), 19.5 cases per 1000 population (95% CI, 16.0 to −23.7), and 28.6 cases per 1000 population (24.3–33.5). None of the cases identified with WHF-screen-positive disease gave a history of previous acute rheumatic fever. Overall, 97.1% of children had normal echocardiograms. The pattern of disease involvement for WHF ‘definite RHD’ and WHF ‘borderline RHD’ cases is presented in *Table 2*.

**Table 1:** Summary statistics from the Echo in Africa project (2014–2018)

	*n* = 5225
Characteristics	
Mean age (SD)	15.0 (2.0)
Female gender, *n* (%)	3439 (65.8)
Prevalence of WHF RHD [weighted no. of cases/1000 (95% CI)]	
WHF ‘definite RHD’	9.1 (6.8–12.1)
WHF ‘borderline RHD’	19.5 (16.0–23.7)
Total WHF RHD	28.6 (24.3–33.5)

CI, confidence interval; RHD, rheumatic heart disease; SD, standard deviation; WHF, World Heart Federation.

**Table 2:** Pattern of WHF echocardiographic valve disease

Definite cases	*n* = 49
(A) ‘Pathological’ MR and at least two morphological features of RHD of the MV, *n* (%)	39 (79.6)
(B) MS with mean gradient >4 mmHg, *n* (%)	0
(C)‘Pathological’ AR and at least two morphological features of RHD of the AV, *n* (%)	7 (14.3)
(D) Borderline disease of both the AV and MV, *n* (%)	3 (6.1)
Borderline cases	*n* = 104
(A) At least two morphological features of RHD of the MV without ‘pathological’ MR or MS, *n* (%)	20 (19.2)
(B) ‘Pathological’ MR, *n* (%)	68 (65.4)
(C) ‘Pathological’ AR, *n* (%)	16 (15.4)

AR, aortic regurgitation; AV, aortic valve; MR, mitral regurgitation; MS, mitral stenosis; MV, mitral valve; RHD, rheumatic heart disease; WHF, World Heart Federation.

Children identified with WHF ‘definite RHD’ Subcategory A (isolated rheumatic MV disease) constituted the majority (*n* = 39/49; 79.6%) of WHF ‘definite RHD’ cases identified in our study cohort. The echocardiographic findings in children identified with WHF ‘definite RHD’ and ‘borderline’ RHD are presented in *Table 3*. The MV criteria that contributed to the majority of WHF ‘definite RHD’ diagnoses were AMVL thickening (57.1%), MV restriction (83.7%), excessive leaflet tip motion (including pseudo-prolapse of the AMVL) (85.7%), and WHF ‘pathological’ MR (85.7%). Chordal thickening was identified in a minority of cases (4%). The aortic valve (AV) criteria that contributed to the majority of WHF ‘definite RHD’ were irregular/focal thickening (18.4%), restricted leaflet motion (20.4%), and WHF ‘pathological’ AR (14.3%). An AV coaptation defect was identified in a single case (2%) with no cases (0%) of AV prolapse identified. Exclusion of the criteria that were least utilized in a WHF ‘definite RHD’ diagnosis (i.e. chordal thickening, coaptation defect and AV prolapse), did not result in a reclassification of any borderline or definite cases.

**Table 3:** Echocardiographic findings in children with WHF ‘definite’- and borderline-RHD

	WHF ‘borderline’ RHD		WHF ‘definite’ RHD	
	(*n* = 104)		(*n* = 49)	
Echocardiographic finding	*n*	%	*n*	%
Morphological MV				
AMVL thickening ≥3 mm	5	4.8	28	57.1
Chordal thickening	2	1.9	2	4
Restricted leaflet motion	14	13.4	41	83.7
Excessive leaflet tip motion	8	7.7	42	85.7
MR				
WHF ‘pathological’ MR	68	65.4	42	85.7
Morphological AV				
Irregular/focal thickening	4	3.8	9	18.4
Coaptation defect	2	1.9	1	2
Restricted leaflet motion	0	0	10	20.4
Prolapse	0	0	0	0
AR				
WHF ‘pathological AR’	16	15.4	7	14.3

AMVL, anterior mitral valve leaflet; AR, aortic regurgitation; AV, aortic valve; MR, mitral regurgitation; MV, mitral valve; RHD, rheumatic heart disease; WHF, World Heart Federation.

Screened cases identified with WHF ‘borderline RHD’ Subcategory B (isolated ‘pathological’ MR) contributed the majority (*n* = 68/104; 65.4%) of WHF ‘borderline RHD’ cases (*Table 2*).

The results of a mechanistic evaluation of MR in WHF ‘borderline RHD’ cases with isolated WHF ‘pathological’ MR are presented in *Table 4*. A mechanistic evaluation of MR in these cases allowed for further classification in 54 children (79.4%). Here, ISS-related MR was identified as the underlying mechanism of MR in 48/68 children (70.5%) with isolated ‘pathological’ MR. In 29/48 children (60.4%), MR was seen to originate from an ISS in the P2/P3 region of the PMVL. The mechanism of MR could not be classified into one of the first four identifiable pre-determined subcategories in 14/68 cases (20.6%) and were classified as indeterminate.

**Table 4:** Mechanism of MR in WHF ‘borderline RHD’ cases with isolated ‘pathological’ MR

	*n* = 68
Mechanism of MR	
ISS, *n* (%)	48 (70.5)
AMVL cleft, *n* (%)	1 (1.5)
MVP/MVPS, *n* (%)	5 (7.4)
Pseudo-prolapse of AMVL, *n* (%)	0 (0)
Indeterminate, *n* (%)	14 (20.6)

AMVL, anterior mitral valve leaflet; ISS, inter-scallop separation; MVP/MVPS, mitral valve prolapse and mitral valve prolapse spectrum.

## Discussion

The findings of this study contribute much-needed data highlighting a heavy burden of latent RHD amongst high-risk South African school children. Furthermore, this study demonstrates that the size of the WHF borderline group, representing the majority of RHD ‘screen-positive’ cases, is driven predominantly by the WHF criteria’s inclusion of cases with isolated WHF ‘pathological’ MR without morphological features of RHD. A mechanistic evaluation of MR, including a focus on identifying the ISS mechanism of MR, substantially reduced the number of WHF ‘borderline RHD’ cases misclassified with RHD.

EIA is the second RHD screening study to be conducted in the Western Cape province of South Africa. In 2015, Engel *et al*.^5^ compared the prevalence of echocardiographic RHD amongst 2720 school children in Bonteheuwel and Langa; two adjacent residential areas within the City of Cape Town municipality. The study reported a WHF ‘definite RHD’ prevalence of 4.8/1000 and an overall subclinical disease prevalence of 20.2/1000, establishing subclinical RHD as an endemic condition amongst select high-risk populations in the Western Cape. The prevalence of subclinical disease reported in the current study supports their data that RHD remains a significant health challenge amongst high-risk children living in this region. Whilst the disease prevalence in our study was not different from that reported by Engel *et al*., there were notable differences in the echocardiographic pattern of WHF ‘definite RHD’ identified in the two studies. In the previous study in Cape Town, concomitant borderline lesions affecting both the MV and AV comprised the majority (76.9%) of reported WHF ‘definite RHD’ cases.^5^ In contrast, the children in our cohort demonstrated more ‘severe’ lesions, so that a definite diagnosis of RHD was typically made with findings from a single valve, whether MV or AV (*Table 2*). The reason for the differences in the reported pattern of valve disease between the studies is not immediately apparent. Various factors may have contributed to these findings that include differences in the acquisition protocol between the two studies as well as the application and interpretation of the WHF criteria. This speaks to the complexity of applying the current criteria consistently despite having a guideline aimed at standardizing assessment and interpretation.

In the current study, ‘pathological’ valve regurgitation was, not unexpectedly, a prominent finding in both AV and MV disease, since a ‘pathological’ functional deficit is a prerequisite for the diagnosis of WHF ‘definite RHD’. In addition, a ‘pathological’ functional deficit in isolation (no associated morphological features) rules a patient into the WHF ‘borderline RHD’ group. The prevalence of WHF ‘borderline RHD’ reported in this study is consistent with the findings published by the majority of large-scale RHD screening studies, where borderline disease constituted between 54% and 88% of echocardiographic RHD.^2–5^^,^^19^^,^^20–22^ There is a concern that the borderline group comprises a heterogeneous group of predominantly non-RHD individuals.^14^^,^^23^ This has important implications for the feasibility of an RHD control programme where the number of screened individuals requiring detailed scans and long-term follow-up has the potential to considerably increase the total screening cost.

The major importance of the implementation of a mechanistic evaluation in the current study was that it showed us that the majority of borderline cases constitute normal spectrum individuals, are not RHD-related and importantly, that this misclassification is brought about primarily by the initial screening focus on MR severity. It is this focus on an MR severity assessment that drives inclusion of isolated mild MR cases into the group. MR, as an isolated finding, without more specific morphological features of RHD, remains a non-specific finding with a number of possible aetiological causes. This is particularly true in the RHD screening setting, considering the often very mild (but WHF ‘pathological’) degrees of MR encountered when screening, which overlap with normal spectrum findings. A frequently employed dogma in RHD screening when dealing with isolated WHF ‘pathological’ MR that demonstrate no morphological RHD features runs as follows: in areas with a high prevalence of RHD, WHF ‘pathological’ MR without a readily identifiable alternative cause, likely represents RHD. Although this seems very reasonable, it does not take into account the high prevalence of another common alternative mechanism of MR. Our research has demonstrated that the major mechanism of MR in cases with isolated ‘pathological’ MR cases is non-rheumatic and related to ISS of the PMVL, a normal finding of the MV.^11^

The question remains what the value of inclusion of isolated ‘pathological’ MR is in the screening context and whether it adds sensitivity to an RHD assessment. It is of critical importance to retain sensitivity when designing a screening test.^24^ To date, it has been very difficult to assess the sensitivity of individual screening criteria due to the absence of an external gold standard test for RHD against which to test such criteria. This makes it impossible to know whether a particular screening criterion improves sensitivity and what the false-positive ‘cost’ of such a strategy is. Another problem that arises with testing the predictive ‘strength’ of an individual criterion (because of the absence of a gold standard test for RHD) is that of incorporation bias. Incorporation bias is unfortunately frequently encountered in RHD research where an individual criterion is assessed against the full WHF criteria which incorporates that same criterion, as validation.^25^^,^^26^ This further decreases certainty about the role individual screening criteria have in the current screening algorithm.

Our assessment of the WHF morphological criteria demonstrated that AMVL thickening, restricted leaflet motion, and excessive leaflet tip motion (including ‘pseudoprolapse’ of the AMVL) were the predominant morphological features constituting WHF ‘definite’ RHD in our study cohort. These findings largely echo those reported by other groups. Recently, Nunes *et al*.^25^ demonstrated that AMVL thickening and excessive leaflet motion were the most predictive for WHF ‘definite RHD’ of the MV. The pattern in our study is similar for AMVL thickening and excessive leaflet tip motion which were present in 57.1% and 85.7% of cases identified with WHF ‘definite’ RHD of the MV, respectively. However, the criterion of restricted leaflet motion, whilst prominent in our cohort (83.7%) was infrequently identified in the derivation cohort described by Nunes *et al*.^25^ There are a number of possible explanations for this. Given the magnitude of the difference in this finding, we consider that true differences in the populations studied are a less likely explanation than the variance in the definitions employed for identifying mild degrees of MV restriction, something we have strictly defined in our study.^17^ Furthermore, when seen in chronic rheumatic disease, the criteria of excessive anterior MV leaflet tip motion and restricted posterior MV leaflet motion tend to describe the same mechanistic observation of ‘pseudoprolapse’ of the AMVL, the most frequently identified mechanism of RHD-related MR.^11^ Here, the finding of excessive leaflet tip motion does not relate to true prolapse but rather describes cases where the tip of the AMVL appears to move excessively relative to a restricted PMVL tip. Importantly, in these cases, the AMVL tip remains well above the MV annulus.^27^^,^^28^ This could explain the differences in the interpretation of restriction or excessive leaflet motion scored in the different studies.

MV chordal thickening was infrequently identified when assessing the MV and together with an AV coaptation defect and prolapse of the AV proved to be redundant in terms of contributing to the diagnosis of WHF ‘definite’ RHD. Regarding MV chordal thickening, it is possible that our screening cohort somehow represents a population with milder disease that has not manifested demonstrable chordal involvement. However, our screening strategy may present another, more plausible explanation. When an EIA case demonstrated apparent chordal thickening in the parasternal long-axis (PSLAX) view, our protocol required that this be confirmed on a detailed apical assessment where specific scanning of the subvalvular was performed. This frequently confirmed that the abnormal chords identified in the PSLAX view were in fact unequivocally normal and that their apparent thickness was related to sidelobe artefact related to the chords running horizontally across the scan plane. This was a frequent finding in normal young individuals with excellent parasternal image quality.

A valid critique of the implementation of the mechanistic assessment, such as implemented in this study, is complexity. Performing a ‘Carpentier-style’ assessment requires training and expertise and may not lend itself to upskilling of healthcare workers and screening in the field.

Since the major reduction in the size of the borderline disease category in this study was achieved by essentially removing misclassified MR cases, it is theoretically possible that a similar reduction could be achieved by refocusing the initial screening on morphology rather than weighting the assessment too heavily on MR severity. This may circumvent the need for an unduly complex mechanistic evaluation in the field and could overcome the potential problem of screening out cases with isolated mild morphological disease.^29^ Further study is needed to determine whether such a ‘morpho-mechanistic’ approach could outperform the current WHF screening methodology and in doing so, reduce the number of cases misclassified with RHD. Such a strategy would have to overcome the difficulty of identifying early morphological features of RHD with a high enough sensitivity and specificity for rheumatic valve involvement in a field where no current gold standard diagnostic test for rheumatic valve involvement exists.

### Limitations

After the first 2 years of EIA, the detailed, second echocardiogram was omitted in screen-negative cases. In the absence of a validating comprehensive study, it is conceivable that some cases of RHD were missed and erroneously misclassified as ‘normal’. However, the data from the first 2 years of comparing HH to comprehensive scans in EIA (data not shown) demonstrated good concordance validating the decision only to perform detailed scans in ‘screen-positive’ or ‘screen-uncertain’ individuals. This has subsequently been validated in other published series^30^ and has become standard in most large-scale RHD screening programmes.

The observation that a mechanistic evaluation in this study reduced the overall number of screen-positive RHD cases that would require further study and potential follow-up suggests a possible cost-saving. However, this will need to be further studied through a formal cost analysis.

Long-term follow-up of EIA-screened participants is essential and is currently underway to describe the natural history of ISS-related MR in cases misclassified with WHF ‘borderline’ RHD.

## Conclusion

Latent RHD remains a significant health challenge in underserved communities within the Western Cape, South Africa. The role of RHD screening as a viable means of secondary prevention of RHD in endemic regions remains controversial in part because the current WHF criteria identify a large, heterogeneous borderline cohort with the potential disease. In this study, the incorporation of a mechanistic evaluation of MR identified ISS—a common, normal feature of the MV, as the underlying mechanism of isolated WHF ‘pathological’ MR in a significant proportion of borderline cases. Further study is needed to investigate the best possible initial screening strategy that will reliably reduce the inclusion of isolated WHF ‘pathological’ MR unrelated to RHD, without decreasing screening sensitivity.

## Lead author biography

Dr Luke David Hunter, MBChB (Stellenbosch University 2011), PhD (Stellenbosch University), is a Medical Doctor and Physician-in-training at Tygerberg Academic Hospital. He is the co-ordinator of the Echo-in-Africa project, a large-scale RHD screening project in the Western Cape, South Africa.

## Supplementary material

Supplementary material is available at *European Heart Journal Open* online.

## Supplementary Material

oeab041_Supplementary_Data
