# Supplementary material for: Screening for subclinical rheumatic heart disease: addressing borderline disease in a real-world setting
Source: Eur Heart J Open. 2021 Dec 27;1(3):oeab041. doi: 10.1093/ehjopen/oeab041 (PMC9242066; doi:10.1093/ehjopen/oeab041)
Supplement: oeab041_Supplementary_Data [file oeab041_supplementary_data.docx]

**Supplementary material**

**Addendum A**

**Reference data* and post hoc weights in the three study areas**

| **Municipality** | Total number of secondary school children | Number of underserved children | Number  of children in schools sampled | Number  of children enrolled | Sampling fraction of schools | Sampling  realisation within schools | weight |
| --- | --- | --- | --- | --- | --- | --- | --- |
| City of Cape  Town | 235400 | 103000 | 15000 | 3474 | 0.146 | 0.232 | 29.65 |
| Drakenstein | 26700 | 18000 | 6000 | 923 | 0.333 | 0.154 | 19.50 |
| Stellenbosch | 27600 | 15000 | 4000 | 828 | 0.267 | 0.207 | 18.12 |

Total 289700 136000 25000 5225 0.184 0.209

*Reference data obtained from annual reports published by the Western Cape Education Department (WCED)
